# Synthesis of Potent Inhibitors of β-Ketoacyl-Acyl Carrier Protein Synthase III as Potential Antimicrobial Agents

**DOI:** 10.3390/molecules17054770

**Published:** 2012-04-25

**Authors:** Yan Liu, Wu Zhong, Rui-Juan Li, Song Li

**Affiliations:** 1 School of Pharmaceutical Engineering, Shenyang Pharmaceutical University, Shenyang 110016, China; Email: liuyanbtnet@163.com (Y.L.); 2 Laboratory of Computer-Aided Drug Design & Discovery, Beijing Institute of Pharmacology and Toxicology, Beijing 100850, China

**Keywords:** tuberculosis, *Mycobacterium tuberculosis* FabH, structure based design, synthesis, alamarBlue™ microassay

## Abstract

*Mycobacterium tuberculosis* FabH, an essential enzyme in the mycolic acid biosynthetic pathway, is an attractive target for novel anti-tubercolosis agents. Structure-based design and synthesis of 1-(4-carboxybutyl)-4-(4-(substituted benzyloxy)phenyl)-1*H*-pyrrole-2-carboxylic acid derivatives **7a–h**, a subset of eight potential FabH inhibitors, is described in this paper. The Vilsmeier-Haack reaction was employed as a key step. The structures of all the newly synthesized compounds were identified by IR, ^1^H-NMR, ^13^C-NMR, ESI-MS and HRMS. The alamarBlue™ microassay was employed to evaluate the compounds **7a–h** against *Mycobacterium tuberculosis* H_37_Rv. The results demonstrate that the compound **7d** possesses good *in vitro* antimycobacterial activity against *Mycobacterium tuberculosis* H_37_Rv (Minimum Inhibitory Concentration value [MIC], 12.5 µg/mL).These compounds may prove useful in the discovery and development of new anti-tuberculosis drugs.

## 1. Introduction

According to data of the World Health Organization [1], Tuberculosis (TB) caused by *Mycobacterium tuberculosis*, is considered to be the most chronic communicable disease in the World especially in Asia and Africa. This situation was made worse by the emergence of multi drug resistant TB (MDR-TB) and the increasing number of HIV-positive TB cases [2]. Worldwide, TB accounts for approximately one-fourth of HIV-related deaths and is the leading cause of death in HIV-infected adults in developing countries [3,4,5], thus an urgent need exists for the development of new antimycobacterial agents with a unique mechanism of action.

The mycobacterial cell wall, which is composed of mycolic acids (α-alkyl-β-hydroxy long chain fatty acids) is known to be important for the growth, survival, and pathogenicity of mycobacteria. Mycobacteria contain both type I (FAS I) and type II (FAS II) fatty acid biosynthetic pathways. FAS is a single multifunctional polypeptide that catalyzes all the reactions in the elongation pathway [6,7]. On the other hand, FAS II system is catalyzed by a series of small, soluble proteins that are each encoded by a discrete gene existing as separate proteins. *Mycobacterium tuberculosis* β-ketoacyl-acyl carrier protein synthase III (*mt*FabH) is a key condensing enzyme responsible for initiation of FAS II fatty acid biosynthetic pathway, and has emerged as an attractive new target for novel anti-tuberculosis agents in recent years. Structure-based design, synthesis of novel inhibitors of *mt*FabH was reported in this paper.

## 2. Results and Discussion

**Figure 1:**
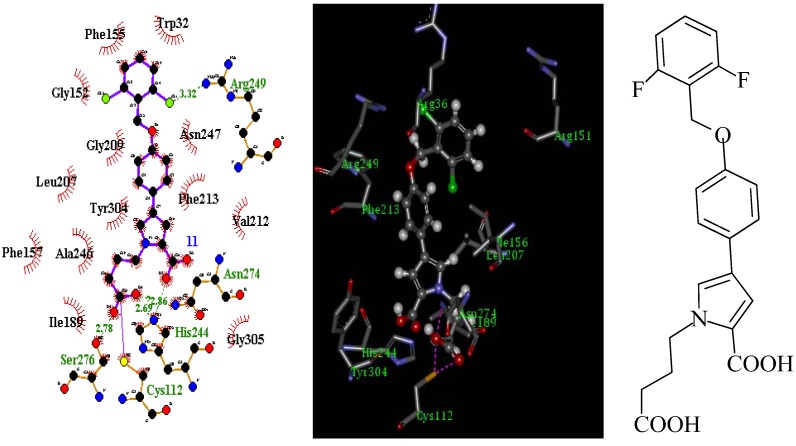
Predicted binding model of compound **7d** in the active site tunnel of *mt*FabH.

We studied all available information about *mt*FabH, including known FabH inhibitors, substrates, and the active site topology from available *mt*FabH crystal structures. According to the structures of the anti-mycobacterial compounds FAS20013, SB418011 and the predicted binding mode of the other known anti-tuberculosis agents [8,9,10,11], we designed a novel scaffold structurs. This structure was predicted to possess improved binding affinity, as shown in Figure 1.

First, a substituted benzyloxy group is required to fill a complementary hydrophobic region at the top of the active site tunnel. Second, the presence of halogen or alkyl substituent is needed in order to form an ionic interaction with the arginine residues (Arg249) at the top of the active site tunnel. Third, the substituted group acidic groups are required to fill the bottom of the active site tunnel, and expected to form direct interaction (such as hydrogen bond) with the catalytic residues (Cys112, His244, Ser276 and Asn274). The scaffold structure itself merely serves as a carrier on which these three binding elements are arranged.

We chose *mt*FabH (PDB ID: 1HZP) [12] as the docking target, and used an automated molecular docking and database screening program DOCK 4.0 [13,14]. A virtual compound library containing 7,560 diverse combinations of substituted benzyloxy and various lengths of fatty acid chains were constructed using Project Library 2.0, a multifunctional combinatorial library construction program [15]. The constructed library was then virtually screened to identify the potential candidate compounds that display favorable binding to the protein active site. The top 500 high free energy score of derivatives from computer screening were carefully investigated. After taking drug-like properties and synthetic accessibility into account, a subset of eight candidate compounds were chosen to be synthesized first. The synthesis of a series of potential *mt*FabH inhibitors are reported in this paper. 

**Scheme 1:**
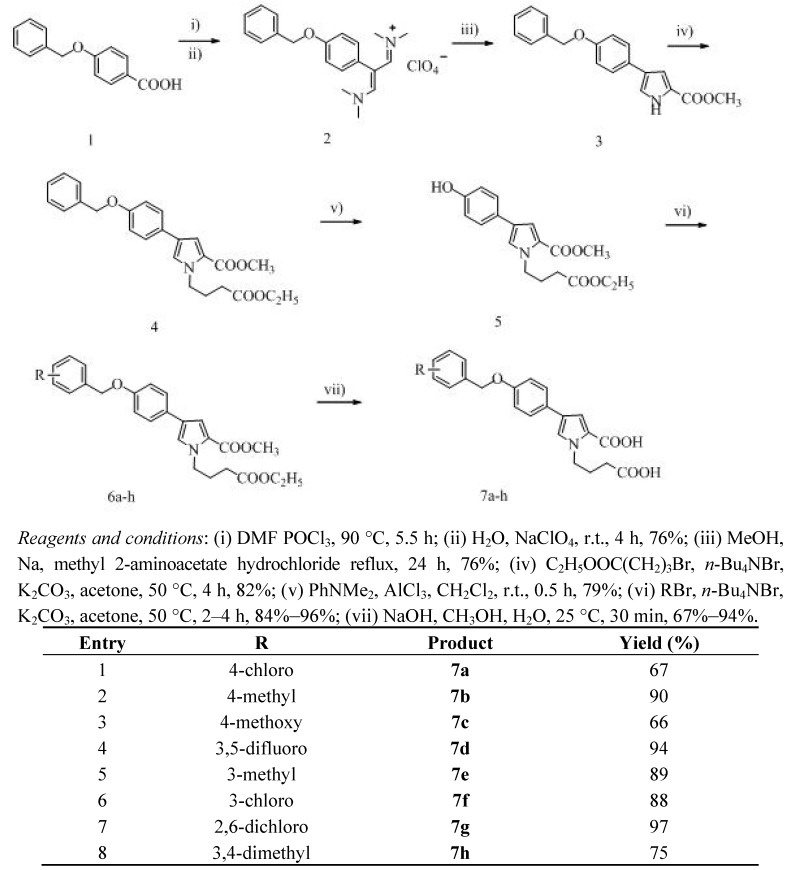
Synthesis of 4-(4-(substituted benzyloxy)phenyl)-1-(3-carboxybutyl)-1*H*-pyrrole-2-carboxylic acid.

As shown in Scheme 1, commercially available 4-benzyloxybenzoic acid (**1**) was used to prepare the vinamidinium salts **2** by the standard Vilsmeier-Haack reaction [16]. When compound **1** was treated with a large excess of Vilsmeier reagent, followed by the addition of sodium perchlorate solution, the 4-benzyloxyphenyl vinamidinium salt was isolated in 76% yield [17]. The cyclization of 4-benzyloxyphenyl vinamidinum salt **2** with methyl 2-aminoacetate hydrochloride in refluxing methanol in the present of sodium methoxide afforded pyrrole-2-carboxylate **3** in 76% yield. Ethyl 4-bromobutanoate with *n*-Bu_4_NBr and anhydrous K_2_CO_3_ were successively added to a stirred solution of **3** in acetone to afford pyrrole-2-carboxylate **4**. Compound **4** was treated with AlC1_3_ and *N,N*-dimethylaniline in CH_2_Cl_2_ to obtain pyrrole-2-carboxylate **5** in 79% yield. To a DMF solution were added compound **5**, substituted benzyl halides and anhydrous K_2_CO_3_ to generate the corresponding pyrrole-2-carboxylates **6a–h**. Compounds **6a–h** were hydrolyzed using sodium hydroxide in a mixture of methanol and water to give compounds **7a–h** in 60%–90% yield.

We determined the antimycobacterial activity of the compounds **7a–h** utilizing a reference strain sensitive to *Mycobacterium tuberculosis* first-line antibiotics (H_37_Rv), obtaining important activity, with a MIC value 12.5 μg/mL. All of the compounds obtained were established unequivocally through analysis of their spectroscopic data. We found no report in the literature on the antituberculosis evaluation of these compounds. The results of their activity are presented in Table 1.

**Table 1:** Antimycobacterial activity (Minimum inhibitory concentration [MIC] μg/mL) of the compounds **7a–h**.

Comp. No.	R	MIC
**7a**	4-chloro	>25
**7b**	4-methyl	>25
**7c**	4-methoxy	>25
**7d**	3,5-difluoro	12.5
**7e**	3-methyl	>25
**7f**	3-chloro	25
**7g**	2,6-dichloro	25
**7h**	3,4-dimethyl	>25
**INH**		0.046

Compounds **7a**, **7b**, **7e** and **7h** have alkyl substituted benzyloxy, which did not demonstrate antituberculosis activity in this study. With regard to the antituberculous activity of the compounds **7f** and **7g**, they demonstrated little such activity against the assayed strains (H_37_Rv), with MIC values ranging between 12.5 and 25 μg/mL. The compound **7d**’s MIC value was found to be 12.5 μg/mL. Further evaluation such as the activity of anti XDR-Mtb strains is still in progress.

## 3. Experimental

### 3.1. Materials and Reagents

All the reagents for synthesis were commercially available and either used without further purification or purified by standard methods prior to use. Melting points were determined using a RY-1 apparatus, and the thermometer was uncorrected. ^1^H-NMR and ^13^C-NMR spectra were measured using an ARX400 multinuclear Fourier transform (FT)-NMR spectrometer (Bruker). Mass spectra were obtained from Micromass ZabSpec 1000 and API3000 instruments.

### 3.2. Synthesis of Starting Compounds ***2–5***

*4-Benzyloxyphenyl* *vinamidinium salt* (**2**). The Vilsmeier-Haack reagent was prepared by slow addition of anhydrous DMF (44 g, 0.6 mol) to phosphorus oxychloride (18.4 g, 0.12 mol) with stirring at 0 °C under a nitrogen atmosphere. The reaction mixture was left at room temperature for 1 h and then 4-benzyloxyphenylacetic acid (**1**, 96.8 g, 0.4 mol) was added and the reaction mixture was stirred for 4.5 h at 90 °C. An aqueous solution (100 mL) of sodium perchlorate (6.2 g, 0.044 mol) was added and the resulting mixture was stirred for 1 h at room temperature. The 4-benzyloxyphenyl vinamidinium salt was isolated in 76% yield.

*4-(4-(Benzyloxy)phenyl)-1H-pyrrole-2-carboxylate* (**3**). A dry, three-necked, round-bottomed flask (500 mL) was equipped with a reflux condenser and magnetic stirrer. Under a nitrogen atmosphere sodium (1.75 g, 0.08 mol) was charged to the flask and then dry methanol (200 mL) was added and the resulting mixture was allowed to react for several minutes while stirring. Methyl 2-aminoacetate hydrochloride (6.4 g, 0.046 mol) was added and then compound **2** (12.5 g, 0.031 mol) was added**.** The resulting mixture was refluxed for 24 h, and the solvent was removed *in vacuo*. To the residue was added water (100 mL) and then the aqueous solution was extracted by EtOAc (3 × 50 mL). Combined organic phase was dried with anhydrous sodium sulfate for 2 h. After filtering out sodium sulfate, the solvent was removed *in vacuo*. The crude product was crystallized twice with the mixture of EtOAc and hexane to give 7.2 g (76% yield) of pure compound **3** as a white solid which had the following properties: Mp 207–208 °C; R*_f_* = 0.2 (hexanes-EtOAc, 3:1). IR (KBr): 3,282, 3,117, 1,678, 1,617, 1,581, 1,570, 1,523, 1,477, 1,465, 1,440, 1,382, 1,297, 1,254, 1,192, 1,180, 1,053, 1,041, 1,026, 994, 926, 809, 769, 728, 692 cm^–1^. ^1^H-NMR (DMSO-d_6_): δ = 11.98 (s, 1H), 7.521–7.543 (d, 2H, *J* = 8.5 Hz), 7.306–7.460 (m, 6H), 7.101–7.111 (t, 1H, *J* = 4 Hz), 6.958–6.980 (d, 2H, *J* = 8.8 Hz), 5.11 (s, 2H), 3.78 (s, 3H). ^13^C-NMR (DMSO-d_6_): δ = 160.8, 156.6, 137.2, 128.4, 127.7, 127.6, 127.4, 125.9, 124.9, 122.4, 120.5, 115.0, 111.6, 69.1, 51.0. ESI-MS *m/z* = 308.1 [M+H]^+^.

*Methyl 1-(4-ethoxy-4-oxobutyl)-4-(4-(benzyloxy)phenyl)-1H-pyrrole-2-carboxylate* (**4**). Ethyl 4-bromo-butanoate (4.73 g, 24.4 mol) with *n*-Bu_4_NBr (10.5 g, 32.6 mmol) and anhydrous K_2_CO_3_ (4.5 g, 32.6 mmol) were successively added to a stirred solution of intermediate **3** (5 g, 16.3 mmol) in acetone (100 mL). The mixture was stirred at 50 °C for 4 h and then the aqueous layer was separated and was acidified with 1 N HCl to pH 4. The aqueous layer was extracted with EtOAc (3 × 50 mL). The combined organic layer was dried (Na_2_SO_4_), filtered, and concentrated under reduced pressure, the remaining residue was purified by column chromatography [silica gel, hexane-EtOAc (v/v) = 10:1] to afford 5.6 g (13.3 mmol, 82% yield) of intermediate **4** as a white solid which had the following properties: Mp 70–71 °C; R*_f_* = 0.3 (hexanes-EtOAc, 8:1). IR (KBr): 3,442, 2,955, 1,728, 1,698, 1,618, 1,567, 1,513, 1,449, 1,392, 1,277, 1,258, 1,192, 1,102, 1,069, 1,041, 1,025, 829, 800, 759, 735, 697 cm^–1^. ^1^H-NMR (DMSO-d_6_): δ = 7.326–7.537 (m, 8H), 7.172–7.177 (d, 1H, *J* = 2 Hz), 6.976–6.997 (d, 2H, *J* = 8.4 Hz), 5.107 (s, 2H), 4.302–4.336 (t, 2H, *J* = 13.6 Hz), 3.989–4.042 (q, 2H, *J* = 21.2 Hz), 3.758 (s, 3H), 2.235–2.272 (t, 2H, *J* = 14.8 Hz), 1.959–1.995 (m, 2H), 1.132–1.168 (t, 3H, *J* = 14.4 Hz). ^13^C-NMR (DMSO-d_6_): δ = 172.1, 160.6, 156.7, 137.2, 128.4, 127.7, 127.6, 126.8, 126.1, 125.8, 122.9, 121.4, 115.1, 114.3, 69.2, 59.9, 51.0, 47.5, 30.5, 26.2, 14.0. ESI-MS *m/z* = 422.2 [M+H]^+^. HRMS-FAB: *m/z* [M+H]^+^ calcd for C_25_H_28_N_1_O_5_: 422.19620; found: 422.19754.

*Methyl 1-(4-ethoxy-4-oxobutyl)-4-(4-(benzyloxy)phenyl)-1H-pyrrole-2-carboxylate* (**5**). To a mixture of compound **4** (5.6 g, 13.2 mmol) and *N,N*-dimethylaniline (12.7 mL, 100 mmol) in anhydrous CH_2_Cl_2_ (30 mL) was added powdered AlC1_3_ (5.2 g, 39.6 mmol) at room temperature. The reaction mixture was stirred for 30 min and then quenched by addition of 1N HCl. The aqueous layer was separated and extracted with EtOAc (3 × 50 mL). The combined organic phase was successively washed with 5% NaHCO_3_ solution and brine, and then dried over anhydrous Na_2_SO_4_ for 1 h. After filtered out Na_2_SO_4_, the organic solvent was removed under reduced pressure. The remaining residue was purified by column chromatography [silica gel, hexanes-EtOAc (v/v) = 4:1] to afford compound **5** 3.48 g (10.5 mmol) as a colorless, oily liquid in 79% yield which had the following properties: R*_f_* = 0.3 (hexanes-EtOAc, 3:1). ^1^H-NMR (DMSO-d_6_): δ = 9.110 (s, 1H), 7.347–7.483 (m, 3H), 7.094–7.099 (d, 1H, *J* = 2 Hz), 6.711–6.740 (m, 2H), 4.312–4.346 (t, 2H, *J* = 13.6 Hz), 3.997–4.050 (q, 2H, *J* = 21.2 Hz), 3.768 (s, 3H), 2.245–2.282 (t, 2H, *J* = 14.8 Hz), 1.967–2.002 (m, 2H), 1.121–1.177 (t, 3H, *J* = 14.4 Hz). ^13^C-NMR (DMSO-d_6_): δ = 172.1, 160.6, 155.8, 125.8, 125.0, 123.4, 121.2, 115.5, 114.1, 59.9, 51.0, 47.5 30.5, 26.3, 14.0. ESI-MS *m/z* = 322.1 [M+H]^+^.

### 3.3. General Procedure for the Synthesis of Compounds ***6a–h***

General Procedure

*Methyl 1-(4-ethoxy-4-oxobutyl)-4-(4-(substituted-benzyloxy)phenyl)-1H-pyrrole-2-carboxylate*. To a solution of intermediate **5** (1 mmol, 0.331 g) and *n*-Bu_4_NBr (0.64 g, 2 mmol) in acetone (20 mL) were added anhydrous K_2_CO_3_ (0.28 g, 2 mmol) and substituted benzyl halides (1.5 mmol). After stirring for 4 h at room temperature, to the reaction mixture 1 N HCl (10 mL) was added. The organic portion was extracted with EtOAc (3 × 20 mL). The combined organic phase was dried for a while and then Na_2_SO_4_ was filtered off and the filtrate was concentrated *in vacuo*. The residue was purified by column chromatography [silica gel, gradient elution with hexane-EtOAc (v/v) = 1:10–1:5] to give the corresponding product.

*Methyl 1-(4-ethoxy-4-oxobutyl)-4-(4-(4-chlorobenzyloxy)phenyl)-1H-pyrrole-2-carboxylate* (**6a**). Colorless, oily liquid; yield: 513 mg (93%); R*_f_* = 0.3 (hexanes-EtOAc, 8:1). ^1^H-NMR (DMSO-d_6_): δ = 7.403–7.558 (m, 6H), 7.176–7.226 (m, 2H), 6.972–7.009 (m, 2H), 5.111–5.152 (t, 2H, *J* = 16.4 Hz), 4.306–4.339 (t, 2H, *J* = 13.2 Hz), 3.988–4.042 (q, 2H, *J* = 21.6 Hz), 3.761 (s, 3H), 2.238–2.276 (t, 2H, *J* = 15.2 Hz), 1.962–2.014 (m, 2H), 1.132–1.168 (t, 3H, *J* = 14.4 Hz). ^13^C-NMR (DMSO-d_6_): δ = 172.5, 161.0, 156.8, 136.6, 132.7, 130.7, 129.8, 127.6, 126.4, 126.1, 121.7, 115.4, 114.7, 110.8, 68.5, 60.2, 51.3, 47.9, 30.9, 26.6, 14.3. ESI-MS *m/z* = 456.2 [M+H]^+^.

*Methyl 1-(4-ethoxy-4-oxobutyl)-4-(4-(4-methylbenzyloxy)phenyl)-1H-pyrrole-2-carboxylate* (**6b**). Colorless, oily liquid; yield: 501 mg (95%); R*_f_* = 0.3 (hexanes-EtOAc, 6:1). ^1^H-NMR (DMSO-d_6_): δ = 7.476–7.498 (m, 3H), 7.319–7.339 (d, 2H, *J* = 8 Hz), 7.170–7.201 (m, 3H), 6.957–6.980 (d, 2H, *J* = 9.2 Hz), 5.050 (s, 2H), 4.302–4.336 (t, 2H, *J* = 13.6 Hz), 3.990–4.043 (q, 2H, *J* = 21.2 Hz), 3.758 (s, 3H), 2.235–2.303 (m, 5H), 1.961–1.996 (m, 2H), 1.133–1.168 (t, 3H, *J* = 14 Hz).^13^C-NMR (DMSO-d_6_): δ = 172.1, 160.1, 156.8, 136.9, 134.1, 128.9, 127.7, 126.7, 125.1, 122.9, 121.4, 115.1, 114.3, 69.1, 59.5, 51.0, 47.5, 30.5, 26.3, 20.7, 14.0. ESI-MS *m/z* = 436.2 [M+H]^+^.

*Methyl 1-(4-ethoxy-4-oxobutyl)-4-(4-(4-methoxybenzyloxy)phenyl)-1H-pyrrole-2-carboxylate* (**6c**). Colorless, oily liquid; yield: 525 mg (96%); R*_f_* = 0.3 (hexanes-EtOAc, 5:1). ^1^H-NMR (DMSO-d_6_): δ = 7.479–7.530 (m, 3H), 7.364–7.386 (d, 2H, *J* = 8.8 Hz), 7.173–7.239 (m, 1H), 6.867–6.981 (m, 4H), 5.015 (s, 2H), 4.304–4.338 (t, 2H, *J* = 13.6 Hz), 3.991–4.044 (q, 2H, *J* = 21.2 Hz), 3.731–3.759 (m, 6H), 2.237–2.274 (t, 2H, *J* = 14.8 Hz), 1.963–2.016 (m, 2H), 1.134–1.169 (t, 3H, *J* = 14.4 Hz). ^13^C-NMR (DMSO-d_6_): δ = 172.1, 160.5, 158.9, 156.8, 129.4, 127.9, 126.7, 126.1, 125.7, 122.9, 115.1, 114.3, 113.8, 113.4, 68.9, 59.8, 55.0, 50.9, 47.6, 30.5, 26.2, 14.0. ESI-MS *m/z* = 452.2 [M+H]^+^.

*Methyl 1-(4-ethoxy-4-oxobutyl)-4-(4-(3,5-difluorobenzyloxy)phenyl)-1H-pyrrole-2-carboxylate* (**6d**). Colorless, oily liquid; yield: 519 mg (94%); R*_f_* = 0.3 (hexanes-EtOAc, 6:1). ^1^H-NMR (DMSO-d_6_): δ = 7.514–7.542 (m, 3H), 7.189–7.201 (m, 4H), 6.994–7.016 (d, 2H, *J* = 8.8 Hz), 5.149 (s, 2H), 4.318–4.352 (t, 2H, *J* = 13.6 Hz), 3.773 (s, 3H), 2.249–2.287 (t, 2H, *J* = 15.2 Hz), 1.978–2.032 (m, 2H), 1.141–1.177 (t, 3H, *J* = 14.4 Hz). ^13^C-NMR (DMSO-d_6_): δ = 172.1, 163.6, 160.6, 156.3, 142.0, 127.2, 126.1, 125.8, 122.8, 121.5, 115.1, 114.3, 110.2, 103.0, 67.8, 59.8, 50.9, 47.6, 30.5, 26.2, 14.0. ESI-MS *m/z* = 458.2 [M+H]^+^.

*Methyl 1-(4-ethoxy-4-oxobutyl)-4-(4-(3-methylbenzyloxy)phenyl)-1H-pyrrole-2-carboxylate* (**6e**). Colorless, oily liquid; yield: 489 mg (93%); R*_f_* = 0.3 (hexanes-EtOAc, 6:1). ^1^H-NMR (DMSO-d_6_): δ = 7.488–7.529 (m, 3H), 7.126–7.291 (m, 5H), 6.972–6.994 (m, 2H), 5.054 (s, 2H), 4.307–4.342 (t, 2H, *J* = 14 Hz), 3.993–4.047 (q, 2H, *J* = 21.6 Hz), 3.762 (s, 3H), 2.239–2.317 (m, 5H), 1.986–2.004 (m, 2H), 1.135–1.171 (t, 3H, *J* = 14.4 Hz). ^13^C-NMR (DMSO-d_6_): δ = 172.1, 160.6, 137.5, 137.1, 128.3, 126.8, 126.1, 125.8, 124.7, 122.9, 121.4, 115.0, 114.3, 69.2, 59.9, 50.9, 47.5, 30.5, 26.2, 20.9, 14.0. ESI-MS *m/z* = 436.2 [M+H]^+^.

*Methyl1-(4-ethoxy-4-oxobutyl)-4-(4-(3-chlorobenzyloxy)phenyl)-1H-pyrrole-2-carboxylate* (**6f**). Colorless, oily liquid; yield: 402 mg (84%); R*_f_* = 0.3 (hexanes-EtOAc, 8:1). ^1^H-NMR (DMSO-d_6_): δ = 7.420–7.552 (m, 6H), 7.173–7.201 (m, 2H), 6.987–7.009 (m, 2H), 5.107–5.149 (t, 2H, *J* = 16.8 Hz), 4.311–4.345 (t, 2H, *J* = 13.6 Hz), 3.993–4.046 (q, 2H, *J* = 21.2 Hz), 3.766 (s, 3H), 2.243–2.280 (t, 2H, *J* = 14.8 Hz), 1.988–2.005 (m, 2H), 1.135–1.171 (t, 3H, *J* = 14.4 Hz). ^13^C-NMR (DMSO-d_6_): δ = 72.5, 160.9, 156.8, 140.1, 133.4, 130.6, 129.7, 128.7, 128.0, 127.5, 126.1, 123.2, 121.7, 115.4, 114.6, 110.7, 68.5, 60.2, 51.3, 47.9, 30.9, 26.6, 14.3. ESI-MS *m/z* = 456.2 [M+H]^+^.

*Methyl 1-(4-ethoxy-4-oxobutyl)-4-(4-(2,6-dichlorobenzyloxy)phenyl)-1H-pyrrole-2-carboxylate* (**6g**). Colorless, oily liquid; yield: 493 mg (95%); R*_f_* = 0.3 (hexanes-EtOAc, 10:1). ^1^H-NMR (DMSO-d_6_): δ = 7.451–7.580 (m, 6H), 7.196–7.201 (d, 1H, *J* = 2 Hz), 7.032–7.054 (d, 2H, *J* = 8.8 Hz), 5.232 (s, 2H), 4.313–4.347 (t, 2H, *J* = 13.6 Hz), 3.995–4.048 (q, 2H, *J* = 21.2 Hz), 3.766 (s, 3H), 2.245–2.282 (t, 2H, *J* = 14.8 Hz), 1.970–2.005 (m, 2H), 1.139–1.174 (t, 3H, *J* = 14 Hz). ^13^C-NMR (DMSO-d_6_): δ = 172.5, 160.9, 158.9, 141.7, 133.8, 129.7, 129.2, 128.0, 127.3, 122.5, 115.3, 114.9, 111.1, 60.2, 51.3, 47.5 30.9, 26.3, 14.0. ESI-MS *m/z* = 490.1 [M+H]^+^.

*Methyl 1-(4-ethoxy-4-oxobutyl)-4-(4-(3, 4-dimethylbenzyloxy)phenyl)-1H-pyrrole-2-carboxylate* (**6h**). Colorless, oily liquid; yield: 449 mg (95%); R*_f_* = 0.3 (hexanes-EtOAc, 6:1). ^1^H-NMR (DMSO-d_6_): δ = 7.483–7.527 (m, 3H), 7.067–7.262 (m, 4H), 6.957–7.020 (m, 2H), 5.003–5.062 (d, 2H, *J* = 23.6 Hz), 4.311–4.339 (t, 2H, *J* = 11.2 Hz), 4.009–4.030 (m, 2H), 3.762–3.801 (m, 3H), 2.203–2.266 (m, 8H), 1.968–2.003 (m, 2H), 1.134–1.169 (m, 3H). ^13^C-NMR (DMSO-d_6_): δ = 172.5, 160.9, 157.2, 136.5, 136.0, 130.6, 134.7, 129.9, 129.7, 129.2, 127.0, 126.4, 126.1, 125.5, 123.3, 121.7, 115.3, 114.6, 68.8, 60.2, 51.3, 47.9, 30.9, 26.6, 19.7, 19.4, 14.3. ESI-MS *m/z* = 450.2 [M+H]^+^.

### 3.4. General Procedure for the Synthesis of Compounds ***7a–h***

General Procedure

*1-(4-carboxybutyl)-4-(4-(substituted benzyloxy)phenyl)-1H-pyrrole-2-carboxylic acid.* To a stirred solution of intermediate **6a–h** in MeOH (50 mL) was added 1 N NaOH (10 mL) at 25 °C. The mixture was stirred at the same temperature for 30 min. The aqueous layer was separated and was acidified with 1N HCl to pH-4. The aqueous layer was extracted with EtOAc (3 × 50 mL). The combined organic layer was dried (Na_2_SO_4_), filtered, and crystallized twice with EtOAc to give pure **7a–h**.

*1-(3-Carboxypropyl)-4-(4-(4-chlorobenzyloxy)phenyl)-1H-pyrrole-2-carboxylic acid* (**7a**). White solid; yield: 313 mg (67%); Mp: 163–164 °C; IR (KBr): 2,958, 1,690, 1,597, 1,512, 1,489, 1,454, 1,434, 1,376, 1,291, 1,178, 1,107, 1,062, 931, 827, 802 cm^–1^; ^1^H-NMR (DMSO-d_6_): δ = 12.193 (s, 2H), 7.464–7.514 (m, 6H), 7.123–7.198 (m, 2H), 6.965–7.001 (m, 2H), 5.109 (s, 2H), 4.290–4.324 (t, 2H, *J* = 13.6 Hz), 2.147–2.185 (t, 2H, *J* = 15.2 Hz), 1.922–1.958 (m, 2H); ^13^C-NMR (DMSO-d_6_): δ = 172.5, 160.9, 156.8, 140.1, 133.4, 130.6, 129.7, 128.7, 128.0, 127.5, 126.1, 123.2, 121.7, 115.4, 114.6, 110.7, 68.5, 60.2, 51.3, 47.9, 30.9, 26.6, 14.3; ESI-MS: *m/z* = 416.5 [M+H]^+^; HRMS-FAB: *m/z* [M+H]^+^ calcd for C_22_H_21_Cl_1_N_1_O_5_: 414.11028; found: 414.10991.

*1-(3-Carboxypropyl)-4-(4-(4-methylbenzyloxy)phenyl)-1H-pyrrole-2-carboxylic acid* (**7b**). White solid; yield: 409 mg (90%); Mp: 186–187 °C; IR (KBr): 2,936, 1,702, 1,616, 1,568, 1,486, 1,291, 1,266, 1,255, 1,203, 1,178, 815, 802 cm^–1^; ^1^H-NMR (DMSO-d_6_): δ = 12.174 (s, 2H), 7.107–7.482 (m, 8H), 6.951–6.973 (d, 2H, *J* = 8.8 Hz), 5.049 (s, 1H), 4.287–4.321 (t, 2H, *J* = 13.6 Hz), 2.304 (s, 3H), 2.145–2.183 (t, 2H, *J* = 15.2 Hz), 1.902–1.975 (m, 2H); ^13^C-NMR (DMSO-d_6_): δ = 173.8, 161.7, 156.6, 137.0, 134.1, 128.9, 127.7, 127.1, 125.7, 125.5, 122.5, 122.5, 115.1, 114.3, 69.1, 47.5, 30.7, 26.4, 20.7; ESI-MS *m/z* = 394.4 [M+H]^+^; HRMS-FAB: *m/z* [M+H]^+^ calcd for C_23_H_24_N_1_O_5_: 394.16490; found: 394.16551.

*1-(3-Carboxypropyl)-4-(4-(4-methoxybenzyloxy)phenyl)-1H-pyrrole-2-carboxylic acid* (**7c**). White solid; yield: 313 mg (66%); Mp: 177–178 °C; IR (KBr): 3,412, 1,749, 1,731, 1,690, 1,612, 1,567, 1,487, 1,402, 1,385, 1,305, 1,284, 1,177, 1,149, 1,094, 832, 810 cm^–1^; ^1^H-NMR (DMSO-d_6_): δ = 12.182 (s, 2H), 7.464–7.486 (m, 3H), 7.367–7.388 (d, 2H, *J* = 8.4 Hz), 7.109–7.114 (d, 1H, *J* = 2.0 Hz), 6.933–6.974 (m, 4H), 5.013 (s, 1H), 4.287–4.322 (t, 2H, *J* = 14.0 Hz), 3.754 (s, 3H), 2.145–2.183 (t, 2H, *J* = 15.2 Hz), 1.902–1.956 (m, 2H); ^13^C-NMR (DMSO-d_6_): δ = 173.8, 161.7, 158.9, 156.7, 129.4, 129.0, 127.0, 125.7, 125.5, 122.6, 122.5, 115.1, 114.2, 113.8, 68.9, 55.1, 47.5, 30.7, 26.4; ESI-MS *m/z* = 410.2 [M+H]^+^; HRMS-FAB: *m/z* [M+H]^+^ calcd for C_23_H_24_N_1_O_6_: 410.15981; found: 410.16113.

*1-(3-Carboxypropyl)-4-(4-(3,5-difluorobenzyloxy)phenyl)-1H-pyrrole-2-carboxylic acid* (**7d**). White solid; yield: 445 mg (94%); Mp: 178–179 °C; IR (KBr): 3,113, 1,732, 1,689, 1,666, 1,615, 1,567, 1,513, 1,451, 1,431, 1,404, 1,383, 1,307, 1,272, 1,255, 1,247, 1,201, 1,094, 1,057, 1,017, 833, 801, 778, 699 cm^–1^; ^1^H-NMR (DMSO-d_6_): δ = 12.203 (s, 2H), 7.488–7.516 (m, 3H), 7.127–7.198 (m, 4H), 6.980–7.002 (d, 2H, *J* = 8.8 Hz), 5.151 (s, 2H), 4.292–4.326 (t, 2H, *J* = 13.6 Hz), 2.148–2.168 (t, 2H, *J* = 15.2 Hz), 1.924–1.960 (m, 2H); ^13^C-NMR (DMSO-d_6_): δ = 173.8, 161.7, 156.1, 127.5, 125.8, 125.6, 122.5, 122.4, 115.1, 114.3, 110.4, 110.4, 110.2, 103.1, 67.8, 47.5, 30.7, 26.5; ESI-MS *m/z* = 416.3 [M+H]^+^; HRMS-FAB: m/z [M+H]^+^ calcd for C_22_H_19_F_2_N_1_O_5_: 416.13041; found: 416.13095.

*1-(3-Carboxypropyl)-4-(4-(3-methylbenzyloxy)phenyl)-1H-pyrrole-2-carboxylic acid* (**7e**). White solid; yield: 381 mg (89%); Mp: 181–182 °C; IR (KBr): 3,113, 2,912, 1,732, 1,689, 1,666, 1,615, 1,567, 1,513, 1,451, 1,431, 1,404, 1,383, 1,307, 1,272, 1,255, 1,247, 1,201, 1,180, 1,094, 1,057, 1,017, 833, 801, 778 cm^–1^; ^1^H-NMR (DMSO-d_6_): δ = 12.217 (s, 2H), 7.474–7.497 (m, 3H), 7.224–7.296 (m, 3H), 7.119–7.149 (m, 2H), 6.964–6.986 (d, 2H, *J* = 8.8 Hz), 5.060 (s, 2H), 4.290–4.323 (t, 2H, *J* = 13.2 Hz), 2.321 (s, 3H), 2.147–2.184 (t, 2H, *J* = 14.8 Hz), 1.903–1.975 (m, 2H); ^13^C-NMR (DMSO-d_6_): δ = 173.8, 161.7, 156.7, 137.5, 137.1, 128.4, 128.3, 128.2, 127.1, 125.7, 125.5, 124.7, 122.5, 122.4, 115.0, 114.2, 113.8, 69.2, 47.5, 30.7, 26.4, 21.0; ESI-MS *m/z* = 394.4 [M+H]^+^; HRMS-FAB: *m/z* [M+H]^+^ calcd for C_23_H_24_N_1_O_5_: 394.16490; found: 394.16600.

*1-(3-Carboxypropyl)-4-(4-(3-chlorobenzyloxy)phenyl)-1H-pyrrole-2-carboxylic acid* (**7f**). White solid; yield: 322 mg (88%); Mp: 173–174 °C; IR (KBr): 2,972, 1,716, 1,668, 1,599, 1,567, 1,513, 1,449, 1,436, 1,379, 1,287, 1,245, 1,215, 1,102, 1,055, 932, 830, 810 cm^−1^; ^1^H-NMR (DMSO-d_6_): δ = 12.217 (s, 2H), 7.424–7.519 (m, 6H), 7.127–7.178 (m, 2H), 6.977–7.000 (m, 2H), 5.110 (s, 2H), 4.291–4.324 (t, 2H, *J* = 13.2 Hz), 2.148–2.186 (t, 2H, *J* = 15.2 Hz), 1.923–1.976 (m, 2H); ^13^C-NMR (DMSO-d_6_): δ = 174.4, 162.3, 156.9, 140.4, 133.6, 130.9, 130.0, 129.0, 128.2, 127.8, 126.6, 126.3, 126.1, 115.7, 114.9, 111.0, 68.8, 48.1, 31.2, 27.0; ESI-MS *m/z* = 414.6 [M+H]^+^; HRMS-FAB: *m/z* [M+H]^+^ calcd for C_22_H_21_Cl_1_N_1_O_5_: 414.11028; found: 414.10996.

*1-(3-Carboxypropyl)-4-(4-(2,6-dichlorobenzyloxy)phenyl)-1H-pyrrole-2-carboxylic acid* (**7g**). White solid; yield: 394 mg (87%); Mp: 208–209 °C; IR (KBr): 3,433, 2,956, 1,706, 1,657, 1,564, 1,510, 1,453, 1,437, 1,383, 1,302, 1,288, 1,263, 1,237, 1,208, 1,190, 1,175, 1,106, 1,055, 1,016, 830, 791 cm^–1^; ^1^H-NMR (DMSO-d_6_): δ = 12.232 (s, 2H), 7.456–7.586 (m, 6H), 7.142–7.148 (d, 1H, *J* = 2.4 Hz), 7.030–7.052 (d, 2H, *J* = 8.8 Hz), 5.230 (s, 2H), 4.300–4.334 (t, 2H, *J* = 13.6 Hz), 2.157–2.195 (t, 2H, *J* = 15.2 Hz), 1.913–1.991 (m, 2H); ^13^C-NMR (DMSO-d_6_): δ = 173.8, 161.7, 156.8, 136.0, 131.8, 131.5, 128.8, 127.6, 125.8, 125.6, 122.5, 122.4, 115.0, 114.3, 65.0, 40.1, 30.7, 26.4; ESI-MS *m/z* = 448.3 [M+H]^+^; HRMS-FAB: *m/z* [M+H]^+^ calcd for C_22_H_20_Cl_2_N_1_O_5_: 448.07130; found: 448.07186.

*1-(3-Carboxypropyl)-4-(4-(3,4-dimethylbenzyloxy)phenyl)-1H-pyrrole-2-carboxylic acid* (**7h**). White solid; yield: 306 mg (75%); Mp: 157–158 °C; IR (KBr): 2,947, 1,716, 1,668, 1,567, 1,512, 1,451, 1,436, 1,364, 1,286, 1,263, 1,178, 1,103, 1,053, 931, 829, 811 cm^–1^; ^1^H-NMR (DMSO-d_6_): δ = 12.228 (s, 2H), 7.465–7.508 (m, 3H), 7.069–7.262 (m, 4H), 6.948–7.012 (m, 2H), 5.013 (s, 2H), 4.293–4.321 (t, 2H, *J* = 11.2 Hz), 2.212–2.271 (m, 6H), 2.146–2.166 (t, 2H, *J* = 8 Hz), 1.922–1.974 (m, 2H); ^13^C-NMR (DMSO-d_6_): δ = 173.8, 161.7, 156.7, 135.7, 134.8, 134.5, 129.4, 128.9, 127.1, 125.7, 125.5, 125.2, 122.5, 122.5, 115.1, 115.0, 114.3, 68.5, 47.5, 30.7, 26.5, 19.4, 19.1; ESI-MS *m/z* = 408.4 [M+H]^+^; HRMS-FAB: *m/z* [M+H]^+^ calcd for C_24_H_26_N_1_O_5_: 408.18055; found: 408.18128.

### 3.5. Antimycobacterial Activity Determination by Fluorometric Microplate Alamar Blue Assay

The methodology was fully described by Collins and Luna-Herrera [18,19], but some modifications were made in the present study. The test was performed in 96-well sterile microplates (Falcon3072; Becton Dickinson, Lincoln Park, NJ, USA). A solution of isoniazid (Sigma cooperation) was prepared in sterile water at a concentration of 5 mg/mL; Pure compounds **7a–h** were dissolved in DMSO at a concentration of 5 mg/mL. The highest concentration wells received 7H9 broth (199 μL).Then the solution of compound (1 μL) was added to the well. The H_37_Rv was grown in 7H9 broth (Difco, Detroit, MI, USA) supplemented with 10% (vol/vol) OADC (oleic acid, albumin, dextrose, catalase; Difco), and 0.05% (vol/vol) Tween 80 (Sigma) at 37 °C in one or two weeks until their turbidity reached McFarland 1 (10^7^ CFU/mL). Simultaneously a 1:20 diluted control was prepared from the bacterial suspension, which represented the growth of 10% of the bacterial population tested. Final testing concentrations are 0.2–0.0125 μg/mL for the isoniazid and from 0.2–25 μg/mL for pure compounds. 

Next, serial two-fold dilutions were prepared and an aliquot of the bacterial suspension (100 μL) was added to these wells. The plates were incubated at 37 °C after 7 days of incubation, one control was developed with 20 μL of 10× alamarBlue™ and 5% Tween80 50 μL solution. The plates were reincubated at 37 °C for 24 h. After this incubation, if the well turned pink, all wells received alamarBlue™ solution and were incubated for an additional 24 h. Fluorescence was measured in a plate fluorometer at a 530-nm excitation wavelength and a 590-nm emission wavelength and Relative fluorescence units (RFU) were recorded. Minimum inhibitory concentration (MIC) was defined as the lowest extract of pure compounds concentration that presented RFU values lower than those presented by the 10% growth control. There was always a correlation between fluorometric and visual observation, *i.e.*, pink wells presented high RFU values. The assay results are summarized in Table 1.

## 4. Conclusions

The present study supports the fact that 1-(4-carboxybutyl)-4-(4-(substituted-benzyloxy)phenyl)-1*H*-pyrrole-2-carboxylic acid derivatives display good activity against resistant *Mycobacterium tuberculosis*. The compound **7d**’s MIC value was found 12.5 μg/mL. Further evaluationg such as the activity of anti XDR-Mtb strains is still in progress. These products may be useful for the development of anti-tuberculosis drugs.

## Supplementary Materials

Supplementary materials can be accessed at: http://www.mdpi.com/1420-3049/17/5/4770/s1.
