# Supplementary material for: Synthesis of Potent Inhibitors of β-Ketoacyl-Acyl Carrier Protein Synthase III as Potential Antimicrobial Agents
Source: Molecules. 2012 Apr 25;17(5):4770–81. doi: 10.3390/molecules17054770 (PMC6268549; doi:10.3390/molecules17054770)

Directions:

The ^{1}H-NMR spectra were measured using a multinuclear Fourier transform (FT)-NMR spectrometer ARX400 (Bruker); Chemical shifts are referenced to internal SiMe4. The serial number in the PDF (like LYM018) is the No. of compound libraries. To help understand, please refer to Table 1

Table 1. The corresponding relation between Compound library No. and Submission No.

Compound library No.	Submission No.
**LYM018**	**7a**
**LYM019**	**7b**
**LYM020**	**7c**
**LYM021**	**7d**
**LYM022**	**7e**
**LYM023**	**7f**
**LYM024**	**7g**
**LYM025**	**7h**

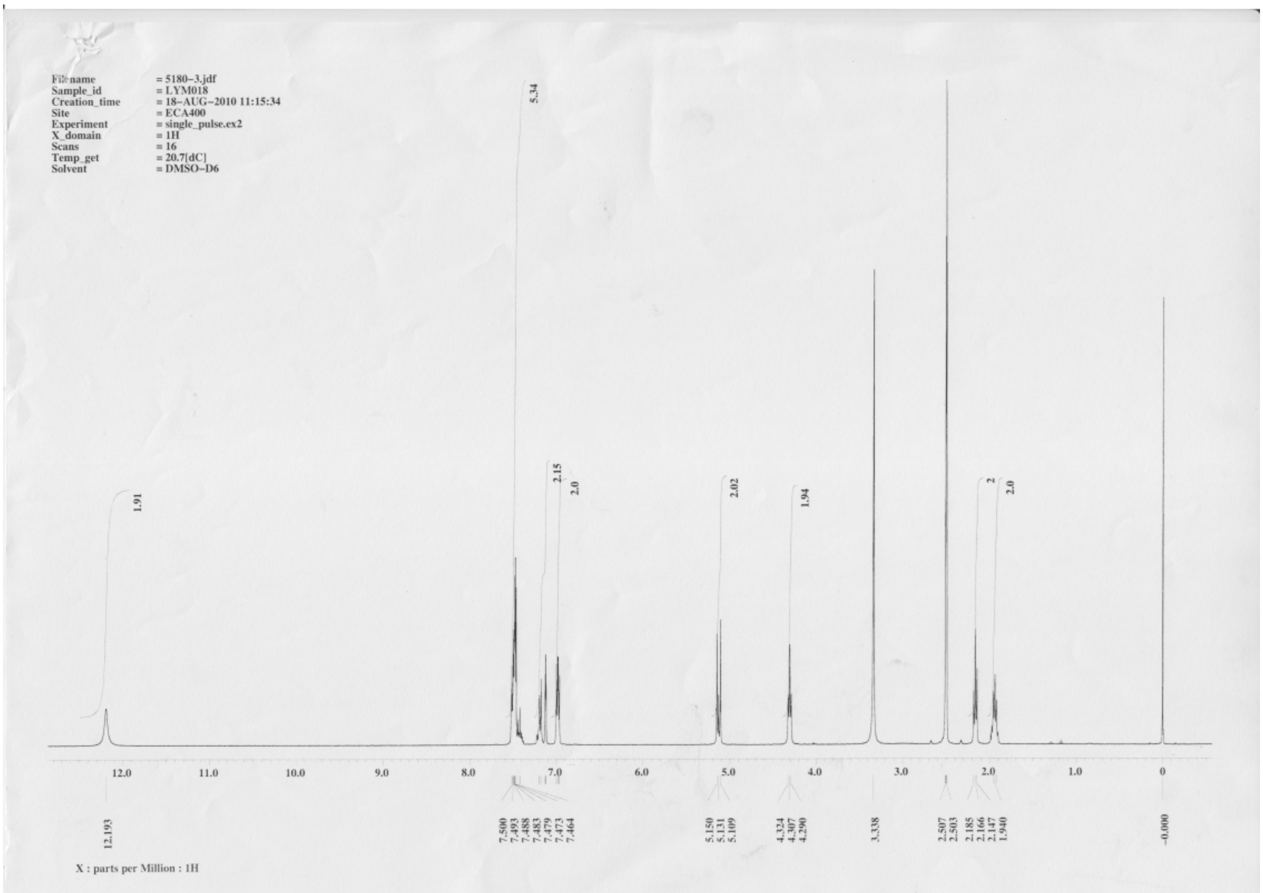

The ^{1}H-NMR spectrum of LYM018

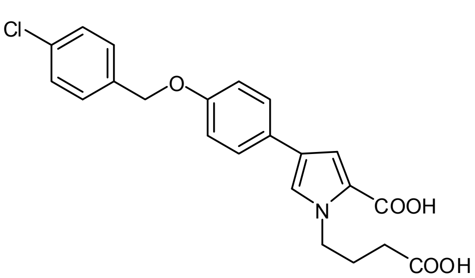

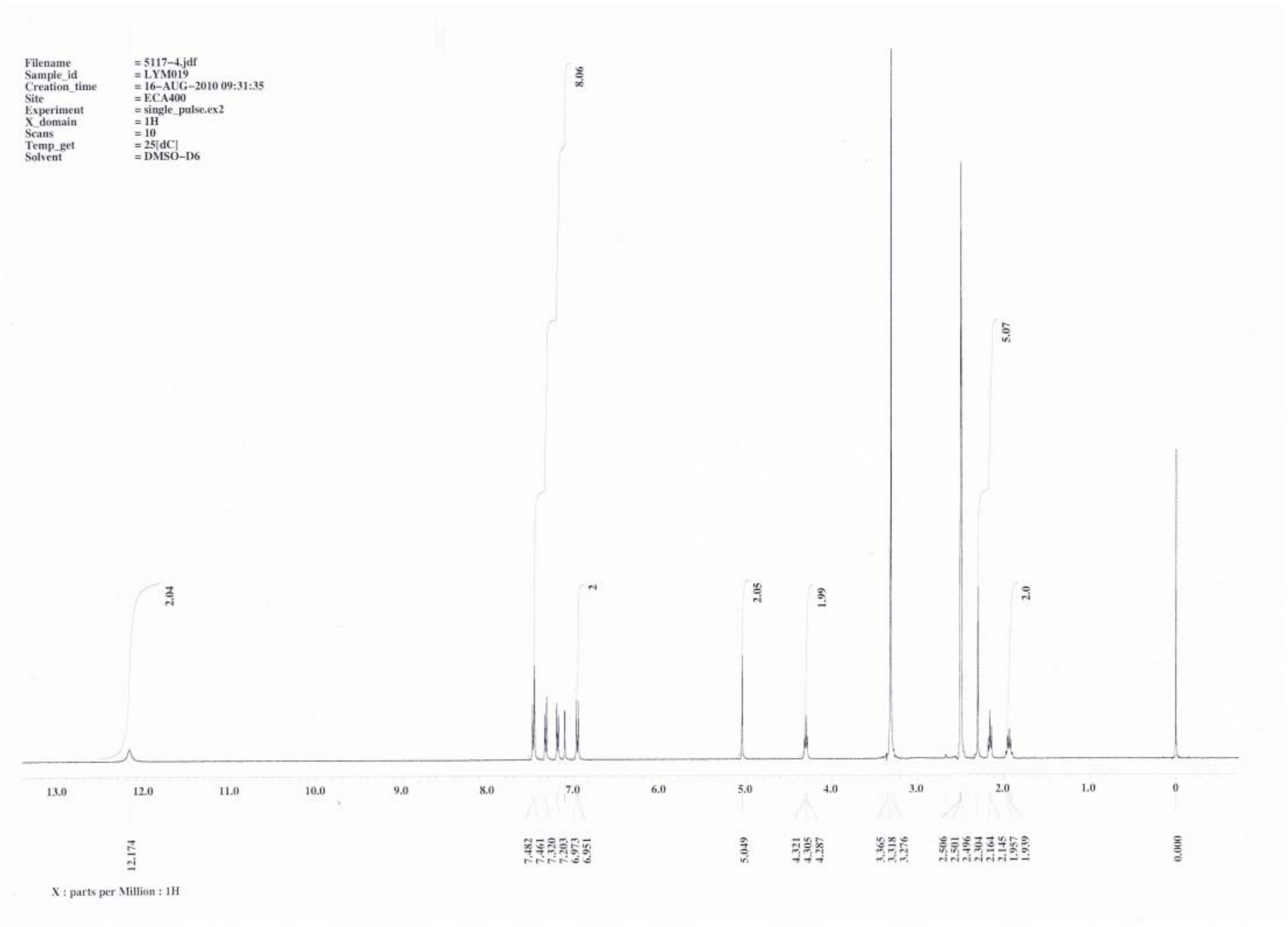

The ^{1}H-NMR spectrum of LYM019

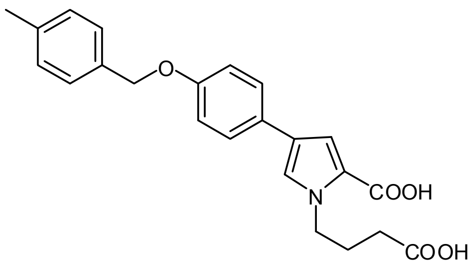

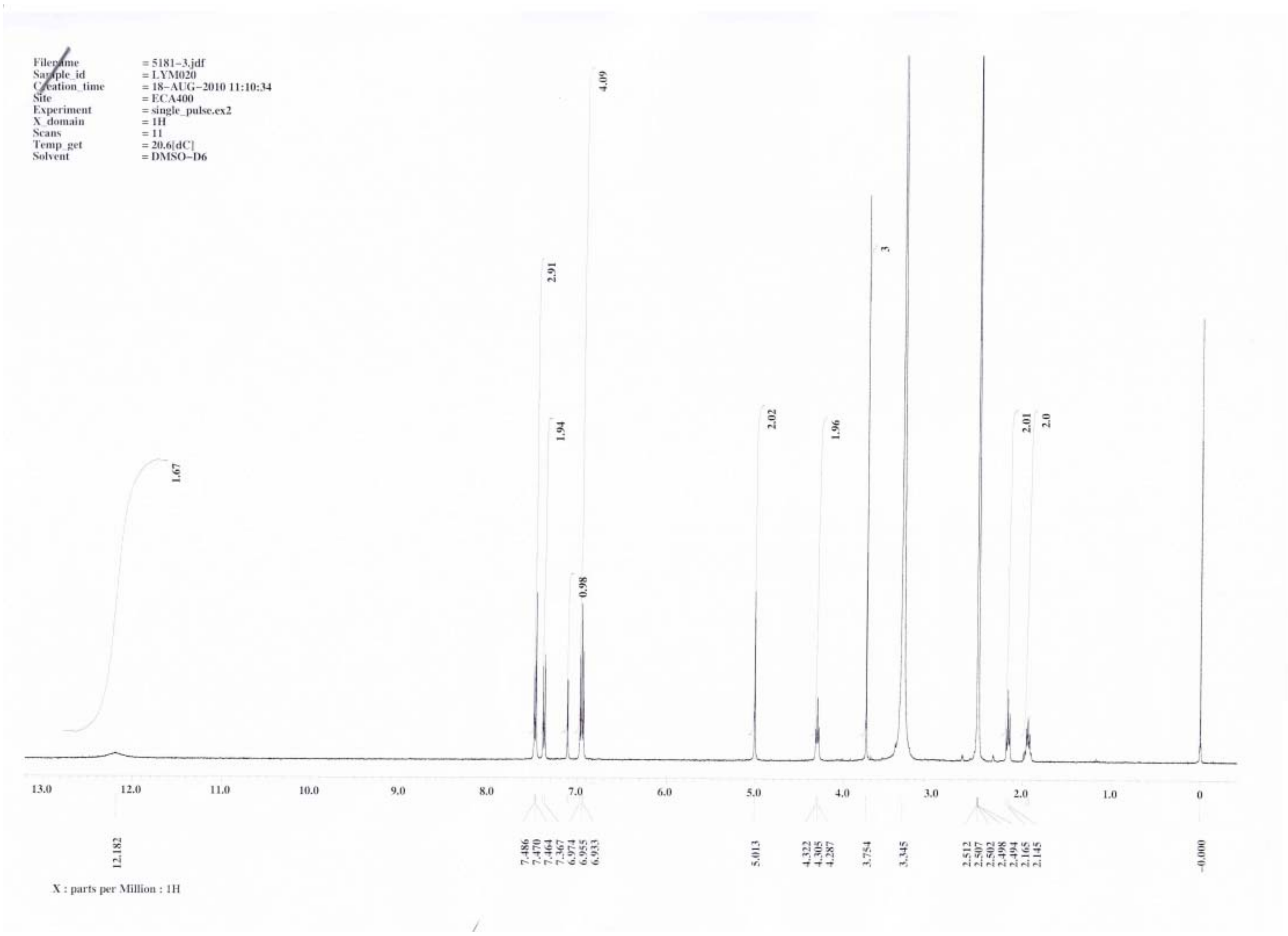

The ^{1}H-NMR spectrum of LYM020

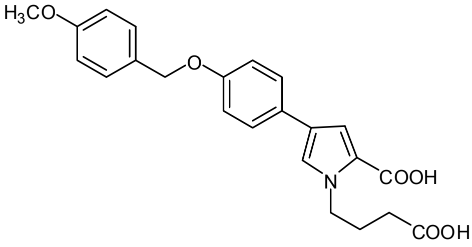

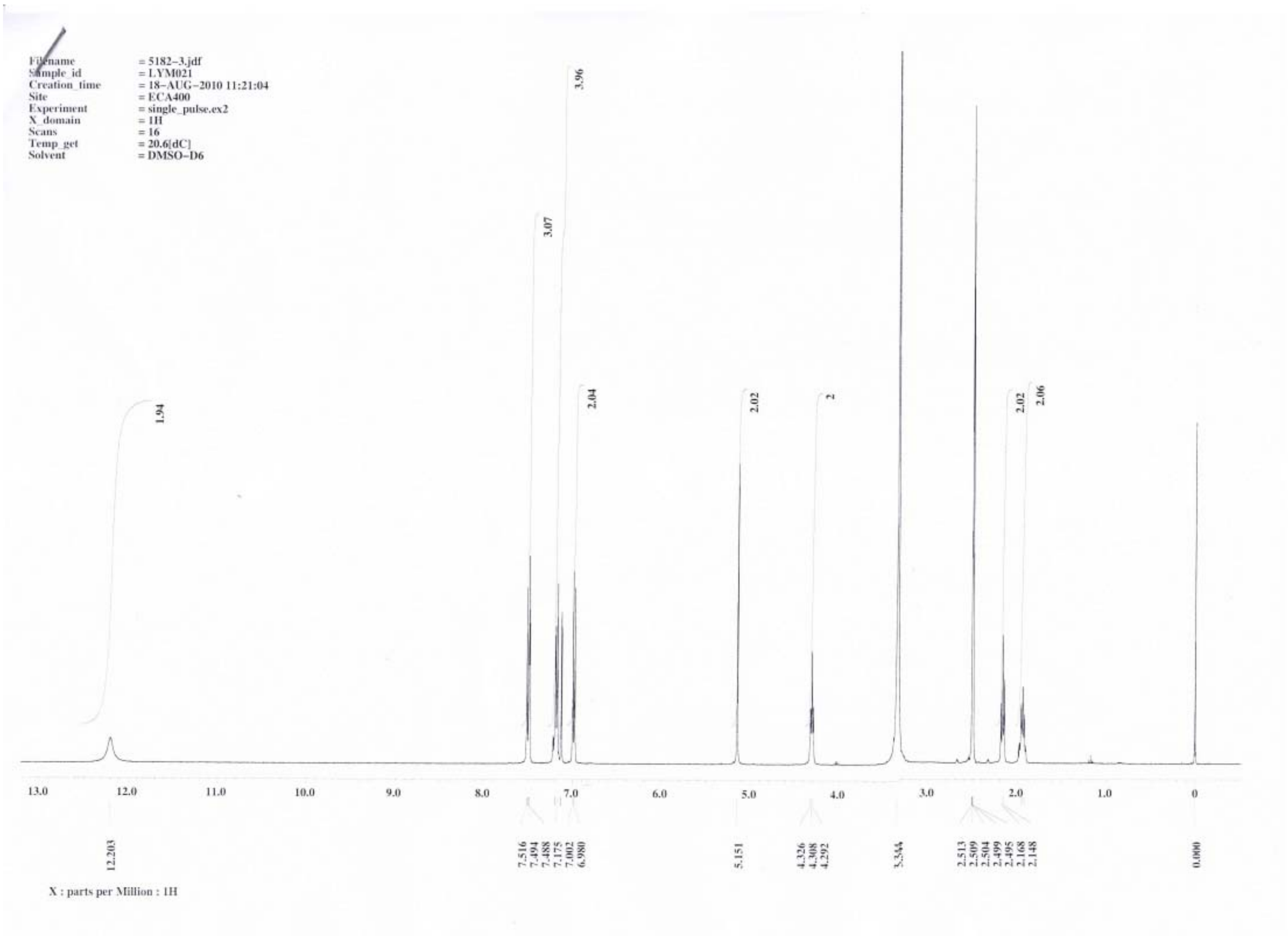

The ^{1}H-NMR spectrum of LYM021

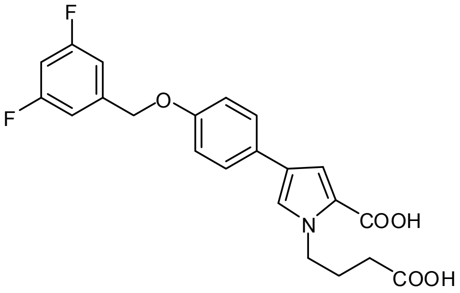

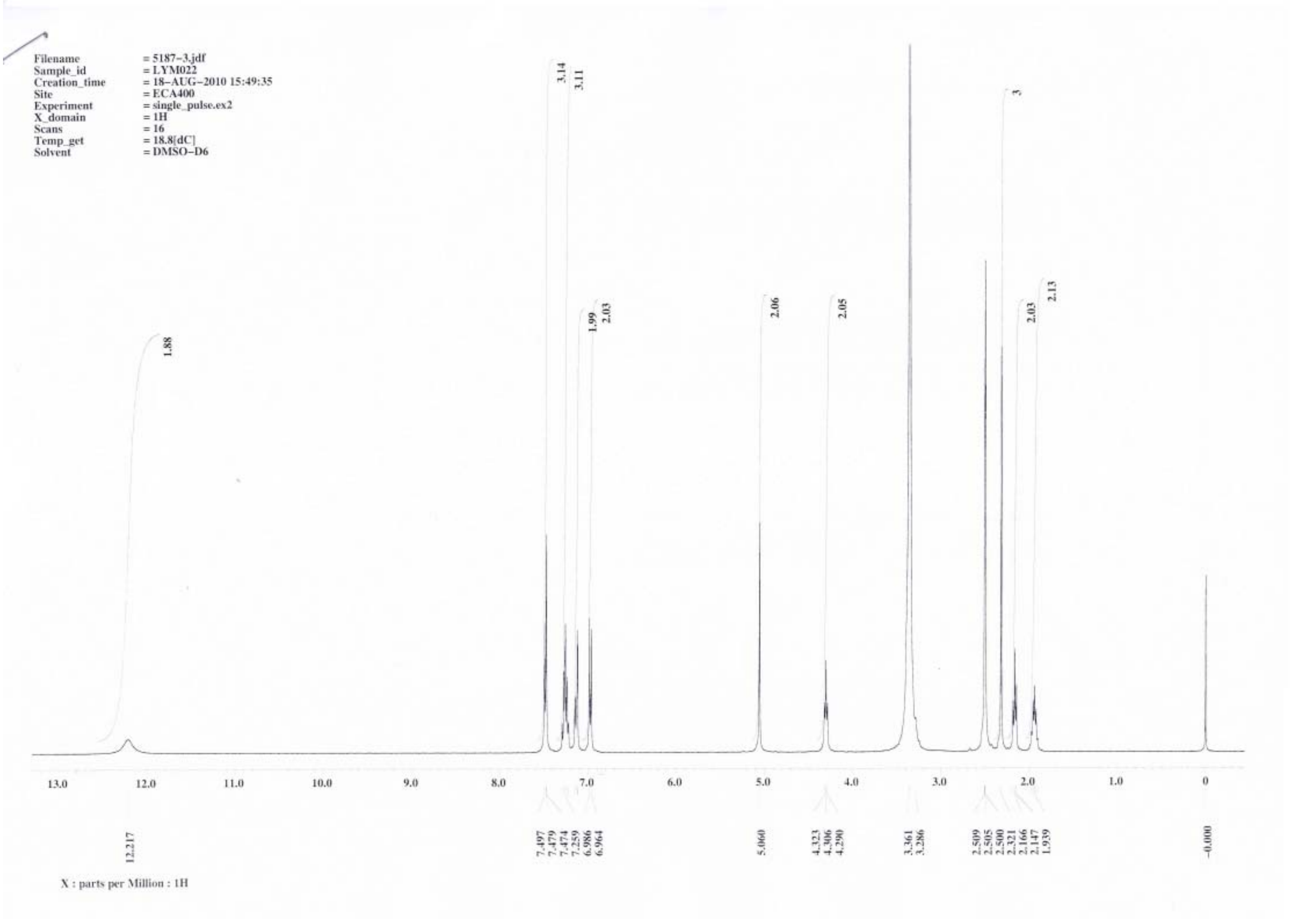

The ^{1}H-NMR spectrum of LYM022

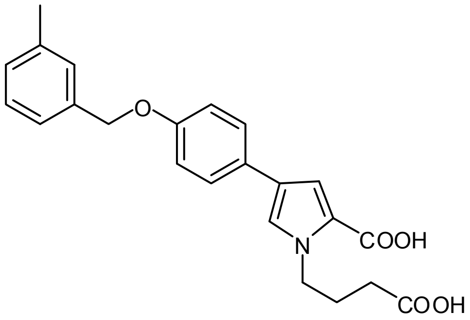

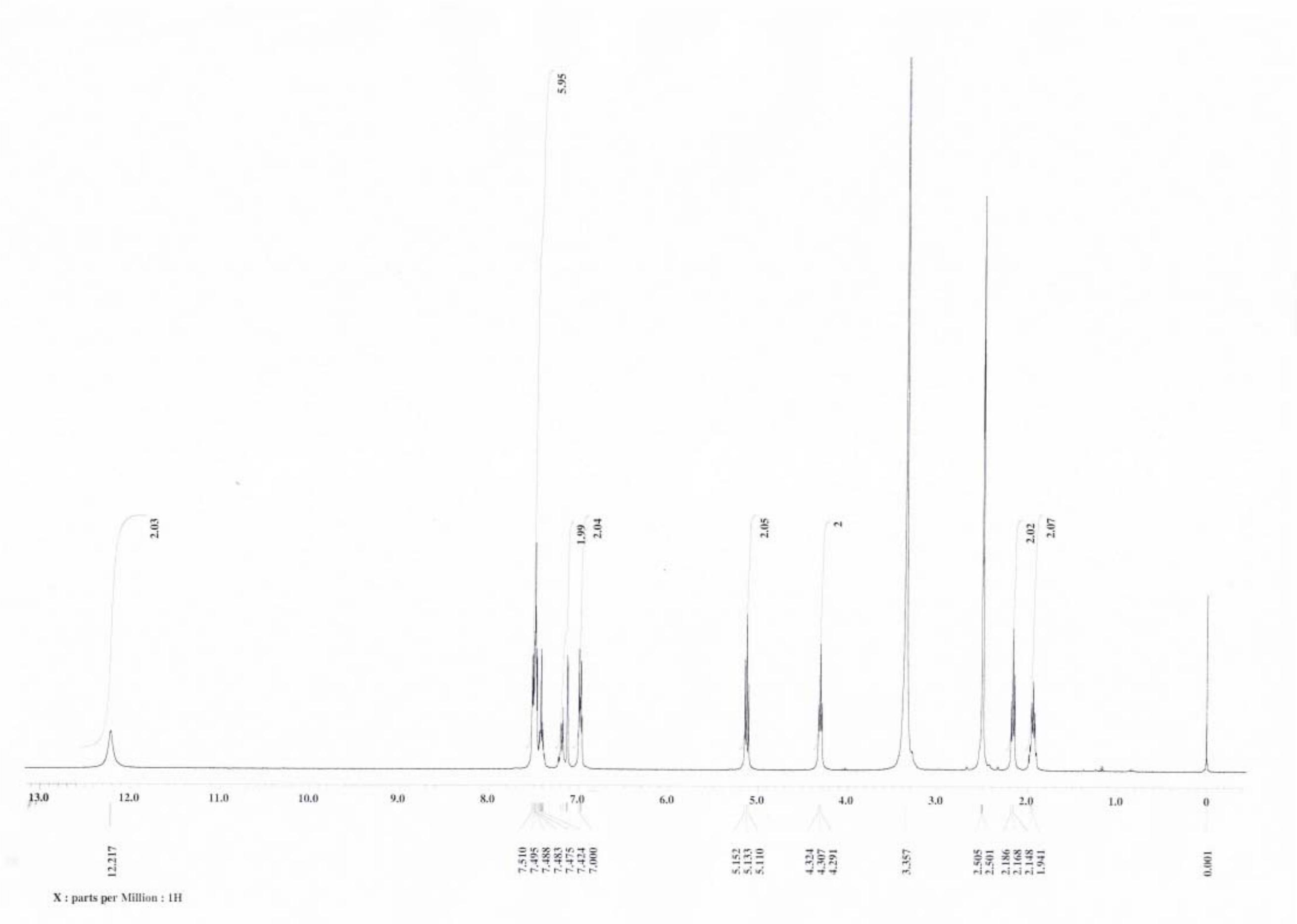

The ^{1}H-NMR spectrum of LYM023

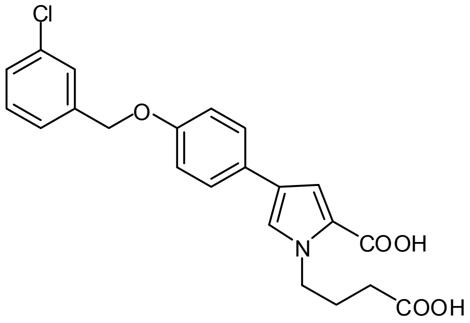

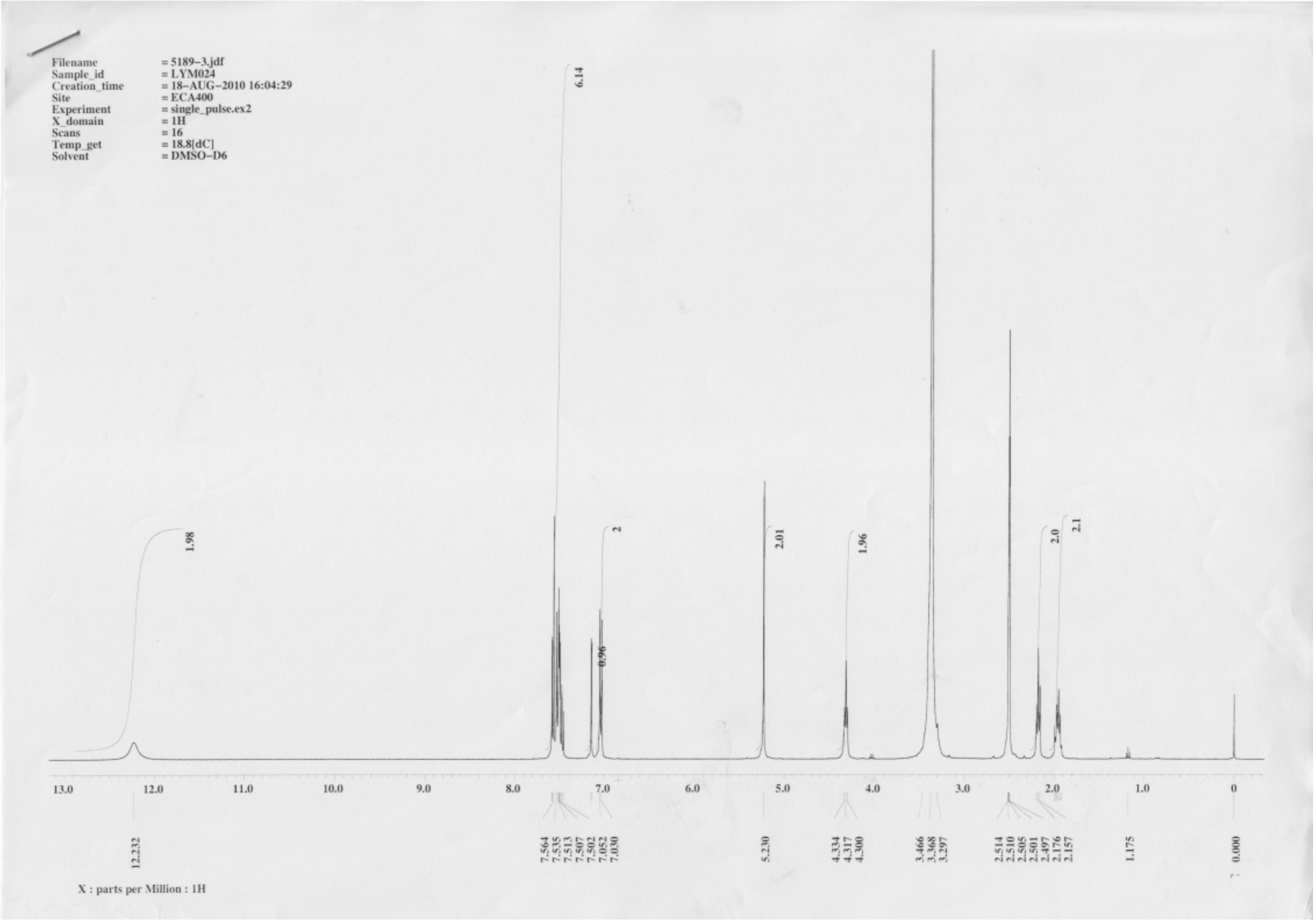

The ^{1}H-NMR spectrum of LYM024

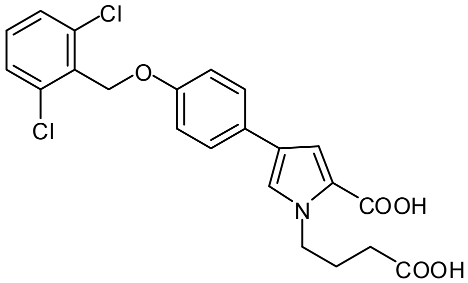

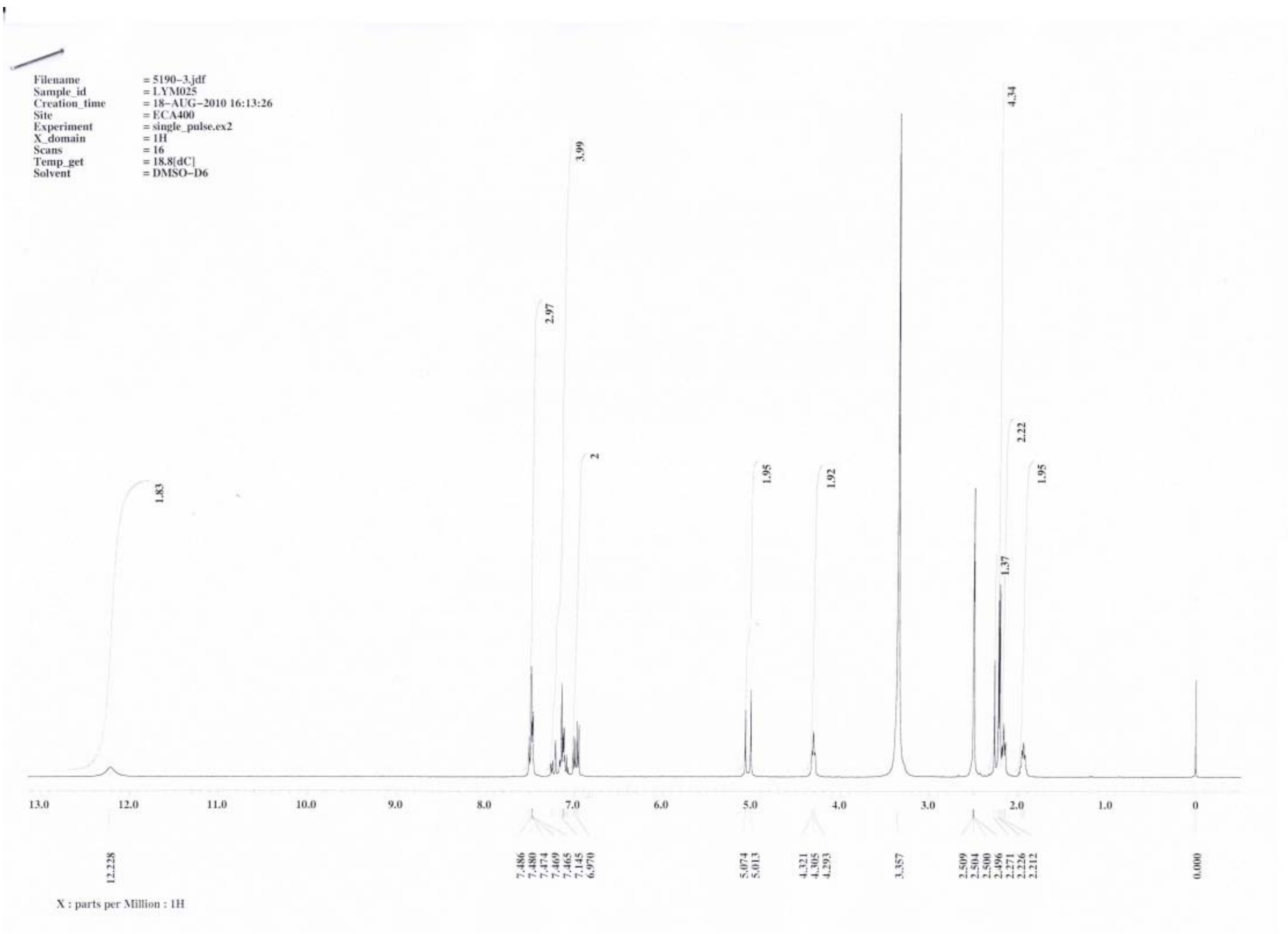

The ^{1}H-NMR spectrum of LYM025

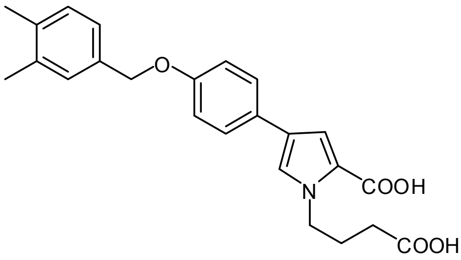

Supplement: Supplementary file 1 [file molecules-17-04770-s001.pdf]
